# Utility of Serum HBV RNA Measurement During Nucleoside/Nucleotide Analog Therapy in Chronic Hepatitis B Patients

**DOI:** 10.3390/ijms262010141

**Published:** 2025-10-18

**Authors:** Keiichi Hiraoka, Masataka Tsuge, Michihiko Kawahara, Hatsue Fujino, Yasutoshi Fujii, Atsushi Ono, Eisuke Murakami, Tomokazu Kawaoka, Daiki Miki, C. Nelson Hayes, Seiya Kashiyama, Sho Mokuda, Shinichi Yamazaki, Shiro Oka

**Affiliations:** 1 Department of Gastroenterology, Graduate School of Biomedical and Health Sciences, Hiroshima University, Hiroshima 734-8551, Japan; khiraoka@hiroshima-u.ac.jp (K.H.); mitumitu@hiroshima-u.ac.jp (M.K.); fujino920@hiroshima-u.ac.jp (H.F.); fujiiyasu@hiroshima-u.ac.jp (Y.F.); atsushi-o@hiroshima-u.ac.jp (A.O.); emusuke@hiroshima-u.ac.jp (E.M.); kawaokatomo@hiroshima-u.ac.jp (T.K.); daikimiki@hiroshima-u.ac.jp (D.M.); oka4683@hiroshima-u.ac.jp (S.O.); 2 Liver Center, Hiroshima University Hospital, Hiroshima 734-8551, Japan; 3 Department of Clinical Oncology, Hiroshima University Hospital, Hiroshima 734-8551, Japan; 4 Section of Clinical Laboratory, Department of Clinical Practice and Support, Hiroshima University Hospital, Hiroshima 734-8551, Japan; kasiyama@hiroshima-u.ac.jp; 5 Division of Laboratory Medicine, Hiroshima University Hospital, Hiroshima 734-8551, Japan; sho-mokuda@hiroshima-u.ac.jp; 6 Department of Clinical Practice and Support, Hiroshima University Hospital, Hiroshima 734-8551, Japan; syamazaki@hiroshima-u.ac.jp

**Keywords:** HBV RNA, HBsAg, HBeAg, HBV DNA, nucleotide/nucleoside analog

## Abstract

Hepatitis B virus (HBV) particles containing HBV RNA are secreted into the blood; these RNA-containing particles are non-infectious byproducts of the replication cycle and are distinct from mature, DNA-containing virions. The proportion of these particles increases during nucleoside/nucleotide analog therapy, but the clinical significance of serum HBV RNA remains unclear. We evaluated longitudinal changes in serum HBV RNA and their association with the antiviral efficacy of nucleoside/nucleotide analog therapy. Eighty-six patients with chronic HBV infection (baseline HBV DNA ≥ 5.0 Log IU/mL and ALT < 500 U/L) treated with entecavir (ETV, N = 80) or tenofovir alafenamide (TAF, N = 6) were included. Serum HBV RNA was quantified using Cobas HBV RNA (RUO) at baseline, week 12, and week 48. Associations with clinical variables and treatment response were analyzed. Baseline HBV RNA correlated with HBsAg, HBV DNA, and hepatitis B core-related antigen. Both HBV DNA and RNA tended to decrease with advanced liver fibrosis. HBV DNA and RNA declines did not differ between HBeAg-positive and -negative patients, but the HBV RNA/DNA ratio at week 48 was significantly higher in HBeAg-positive cases (*p* < 0.001). Patients with baseline ALT ≥ 100 U/L showed significantly lower RNA levels at weeks 12 and 48 (*p* = 0.004, *p* < 0.001). While HBV DNA decline was similar between ETV and TAF (*p* = 0.076), RNA decline was significantly greater with TAF at week 12 (*p* = 0.027). Serum HBV RNA reflects intrahepatic viral replication and may not be influenced by fibrosis progression. HBV RNA decline during nucleoside/nucleotide analog therapy differed between ETV and TAF, suggesting drug-specific effects on viral RNA dynamics.

## 1. Introduction

Hepatitis B virus (HBV) infection is a serious global health problem. Although the number of cases of new HBV infection has been declining globally due to a universal vaccination program, 1.2 million new HBV infections still occur each year, and 254 million people remain chronically infected [1]. Chronic HBV infection often leads to the development of chronic hepatitis, liver cirrhosis, and hepatocellular carcinoma, and the incidence of hepatocellular carcinoma in chronically infected individuals is 22.4-fold higher than in uninfected healthy individuals [2]. Once HBV infects human hepatocytes, it becomes difficult to eliminate because mature HBV DNA is sequestered in the nucleus in the form of a covalently closed circular DNA (cccDNA) mini-chromosome, and HBV DNA sometimes becomes integrated into human chromosomes [3,4,5,6]. CccDNA functions as the template for viral replication and is used to transcribe viral RNAs for the production of HBV-related proteins and pregenomic RNA. In contrast, while double-stranded HBV DNA may become integrated into the host genome, integration often occurs randomly via host DNA repair mechanisms and is not essential for viral replication. To suppress viral replication and prevent progression of liver disease in patients with chronic hepatitis B (CHB) infection, antiviral therapies using interferon and/or nucleoside/nucleotide analogs (NAs) are administered [7,8,9]. Current NA antiviral therapies, such as entecavir (ETV) or tenofovir, can strongly suppress viral replication, and serum HBV DNA levels in most CHB patients fall below the limit of detection within one year. However, even when serum HBV DNA level is maintained at unmeasurable levels as a result of NA therapy, the production of HBV-related proteins, such as HBsAg, may continue due to transcription from cccDNA and HBV genomic integration [10] and may contribute to promotion of hepatocarcinogenesis during NA therapy [11]. Therefore, it is important to monitor HBV replication in human hepatocytes during antiviral therapy, and effective viral markers for evaluating HBV replication in the liver are needed [12].

In HBV-infected hepatocytes, HBV pregenomic RNA (pgRNA) is transcribed from cccDNA, exported from the nucleus, and encapsidated into HBV core particles in the cytoplasm. Most pgRNAs are reverse-transcribed into negative-stranded genomic DNA in the core particle before being transformed into partially double-stranded HBV DNA genomes before secretion [13,14]. However, a subset of pgRNA-encapsidating particles are secreted without completing reverse transcription, especially under NA therapy. This reflects the dual functions of the HBV RNA epsilon stem-loop structure in both priming of reverse transcription as well as packaging of pgRNA. NA therapy blocks elongation, but epsilon-mediated packaging continues, resulting in defective RNA-filled particles as by-products of aborted replication rather than a programmed pathway [15]. Excessive accumulation of particles containing HBV RNA might occur in hepatocytes, leading to the release of HBV particles containing HBV RNA into the blood [16,17,18,19]. In our previous study, the presence of HBV RNA particles in the serum was significantly associated with the development of antiviral resistance [16]. Therefore, monitoring of relative serum HBV DNA and HBV RNA levels might be a useful method for predicting reactivation of chronic hepatitis B after discontinuation of NA therapy [20]. Unfortunately, while we did not have access to commercial products for directly measuring HBV RNA at that time, and the method for measuring serum HBV RNA was complex, we were nonetheless able to measure HBV DNA and HBV RNA levels in our previous studies.

Recently, a commercial product to measure serum HBV pgRNA levels has become available, and several studies have indicated that HBV pgRNA can be detected in the serum of CHB patients who have never experienced antiviral therapy, thereby serving as a marker for viral replication [21,22]. To clarify the usefulness of serum HBV RNA level for evaluating NA therapy, we measured serum HBV RNA levels before and during ETV therapy. We determined that serum HBV RNA levels predicted HBeAg seroconversion and early HBV DNA reduction in HBeAg-positive patients [23]. However, the association between serum HBV RNA level and liver fibrosis stage has not been clarified, nor has the effect of different NA therapies on HBV RNA alteration. To clarify these points, we compared antiviral effects using several HBV-related markers, including HBV RNA in CHB patients treated with ETV or TAF.

## 2. Results

### 2.1. Comparison of Initial Values Between the ETV and TAF Groups

Baseline characteristics at the initial visit are shown in Table 1. The median age of enrolled CHB patients was 48 years, and 63 of 86 patients were male. Forty-seven patients were positive for HBeAg. Eighty patients were treated with ETV, while the remaining 6 patients were treated with TAF. There was no significant difference between the ETV and TAF groups for any factor except liver fibrosis stage (*p* = 0.007).

### 2.2. Correlations Between HBV RNA Level and Other HBV-Related Markers at Baseline

**Figure 1:**
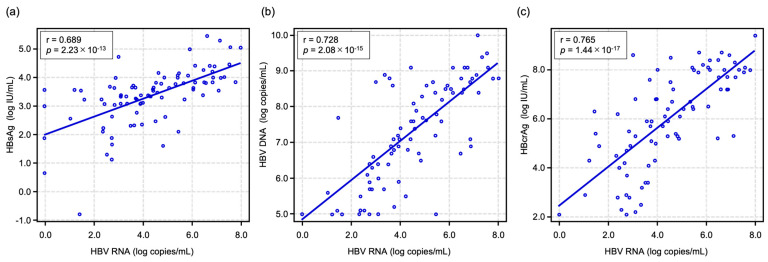
Associations between serum HBV RNA and HBV-related markers before NA therapy. Scatter plots showing the relationships between HBV RNA and (**a**) HBsAg, (**b**) HBV DNA, and (**c**) HBcrAg in patients with chronic hepatitis B infection. Statistical analyses were performed using Spearman’s rank correlation test. HBV, hepatitis B virus; NA, nucleoside analog; HBsAg, hepatitis B surface antigen; HBcrAg, hepatitis B core-related antigen.

First, we assessed baseline correlations between HBV RNA and other HBV markers to evaluate whether serum RNA correlated with replicative and transcriptional activity at the start of the study. As shown in Figure 1, HBV RNA level was significantly correlated with HBsAg (r = 0.689, *p* = 2.23 × 10^−13^), HBV DNA (r = 0.728, *p* = 2.08 × 10^−15^), and HBcrAg (r = 0.765, *p* = 1.44 × 10^−17^).

**Figure 2:**
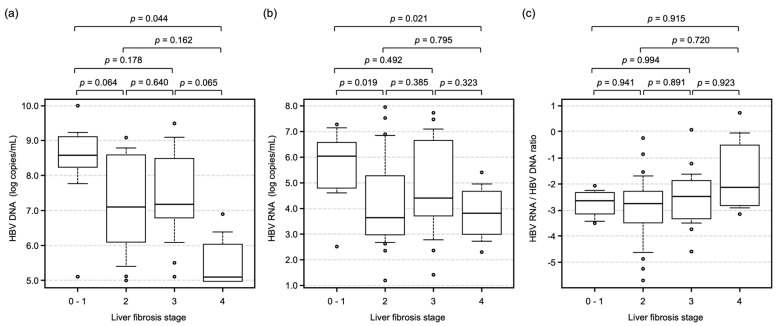
Associations between HBV-related markers and liver fibrosis stage. Box plots showing (**a**) serum HBV DNA, (**b**) serum HBV RNA, and (**c**) the HBV RNA/HBV DNA ratio with respect to fibrosis stages (F0–1, F2, F3, F4). Boxes indicate the median and interquartile range (IQR); whiskers denote the 10th–90th percentiles. Statistical analyses were performed using the Kruskal–Wallis test. Post hoc pairwise comparisons were conducted with the Steel–Dwass procedure for all pairs.

To analyze the impact of liver fibrosis progression, HBV DNA and RNA levels were compared at each fibrosis stage. Although HBV DNA and RNA levels decreased significantly following liver fibrosis progression (Figure 2a,b), the HBV RNA/HBV DNA ratio was not altered, regardless of liver fibrosis grade (Figure 2c).

### 2.3. Comparison of the Alterations of HBV-Related Markers Between HBeAg-Positive and -Negative Patients During NA Therapy

**Figure 3:**
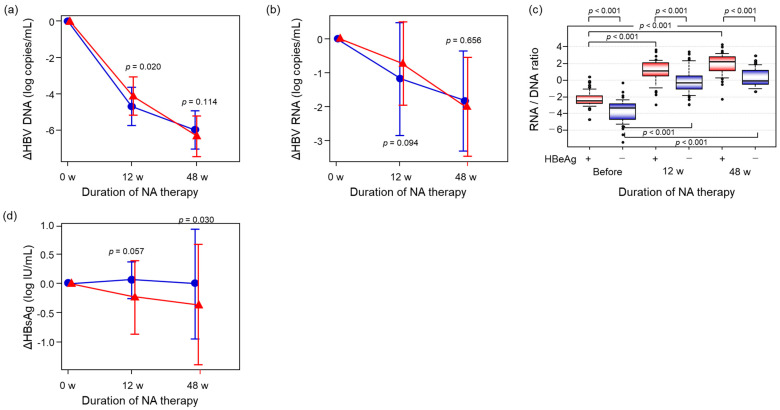
Dynamics of HBV-related markers during NA therapy in comparison between HBeAg-positive and -negative patients. Red lines represent the HBeAg-positive group, while blue lines indicate the HBeAg-negative group. (**a**,**b**) The decline in HBV DNA and HBV RNA levels from baseline (before NA therapy) was calculated and compared between the HBeAg-positive (N = 47) and the HBeAg-negative group (N = 39). (**c**) The RNA/DNA ratio at each time point was compared between the HBeAg-positive and HBeAg-negative groups. (**d**) The decline in HBsAg levels from baseline (before NA therapy) was calculated and compared between the HBeAg-positive and HBeAg-negative groups. All statistical analyses were performed using the Mann–Whitney U test, except (**c**), which used the Kruskal–Wallis test. Post hoc pairwise comparisons were conducted with the Steel–Dwass procedure. HBeAg, hepatitis B e antigen; HBsAg, hepatitis B surface antigen.

HBeAg status is indicative of discrete phases of chronic HBV infection, characterized by varying replication and immune states. To clarify the impact of HBeAg status, patients were divided into two groups based on the presence of HBeAg (HBeAg-positive: N = 47, HBeAg-negative: N = 39). Although the rate of decline of both HBV DNA and HBV RNA by 12 weeks in the HBeAg-negative group was higher than in the HBeAg-positive group (*p* = 0.020, *p* = 0.094, respectively), the level did not differ between the two groups after 48 weeks of treatment (*p* = 0.114, *p* = 0.658, respectively) (Figure 3a,b). To identify the impact of NA therapy on HBV reverse transcription during HBV replication [24], we calculated the ratio of HBV RNA to HBV DNA (RNA/DNA ratio). The RNA/DNA ratio prior to NA therapy in the HBeAg-positive group was significantly higher than that in the HBeAg-negative group (*p* < 0.001), and the difference was magnified after 48 weeks of treatment (*p* < 0.001) (Figure 3c). On the contrary, HBsAg level declined significantly more in the HBeAg-positive group than the HBeAg-negative group at 48 weeks (*p* = 0.030) (Figure 3d).

### 2.4. Differences in the Alterations of HBV-Related Markers Between the ETV and TAF Groups

**Figure 4:**
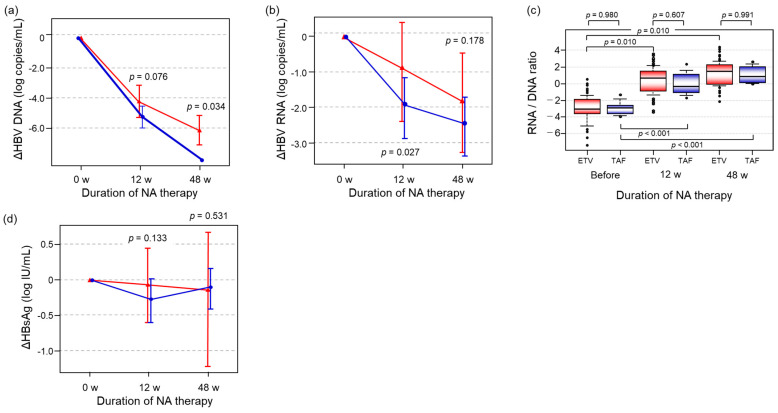
Dynamics of HBV-related markers during NA therapy in comparison between the ETV and TAF groups. Red lines indicate the ETV group, while blue lines indicate the TAF group. (**a**,**b**) HBV DNA and HBV RNA decline from baseline (before NA therapy) was calculated and compared between the ETV (N = 80) and TAF groups (N = 6). (**c**) The RNA/DNA ratio at each time point was compared between the ETV and TAF groups. (**d**) HBsAg decline from baseline was calculated and compared between the ETV and TAF groups. All statistical analyses were performed using the Mann–Whitney U test, except (**c**), which used the Kruskal–Wallis test. Post hoc pairwise comparisons were conducted with the Steel–Dwass procedure. NA, nucleoside analog; HBeAg, hepatitis B e antigen; HBsAg, hepatitis B surface antigen; HBsAg, hepatitis B surface antigen; ETV, entecavir; TAF, tenofovir alafenamide.

In view of the pharmacological differences between ETV and TAF, we evaluated whether RNA and DNA suppression patterns diverged by regimen. The study patients were divided into two groups based on the type of NA received (ETV group: N = 80; TAF group: N = 6). In this cohort, 80% and 83% of enrolled patients in the ETV and TAF groups were infected with HBV genotype C, respectively, and only 2 patients in the ETV group were infected with genotype B (Table 1). Although there was no difference between the two groups with respect to HBV DNA and HBV RNA titers prior to NA therapy (Table 1), HBV DNA decline in the TAF group was significantly greater than in the ETV group (*p* = 0.034) (Figure 4a). Interestingly, although HBV RNA decline in the TAF group after 12 weeks of treatment was significantly higher than that in the ETV group after 12 weeks of treatment (*p* = 0.027), no difference in HBV RNA decline was observed after 48 weeks of treatment *p* = 0.178) (Figure 4b). In comparison of the RNA/DNA ratio before NA therapy between the ETV and TAF groups, ratios in both the ETV and TAF groups were significantly increased from baseline to 12 weeks of NA therapy (*p* < 0.001), and the ratios were maintained until 48 weeks of treatment (Figure 4c). However, the ratios at each time point did not differ between treatment groups (*p* = 0.980 before treatment, *p* = 0.607 at 12 weeks of treatment, and *p* = 0.991 at 48 weeks of treatment). When we compared HBsAg alteration, there was no difference between the two groups (Figure 4d).

### 2.5. The Impact of ALT Level on the Alteration of HBV-Related Markers During NA Therapy

**Figure 5:**
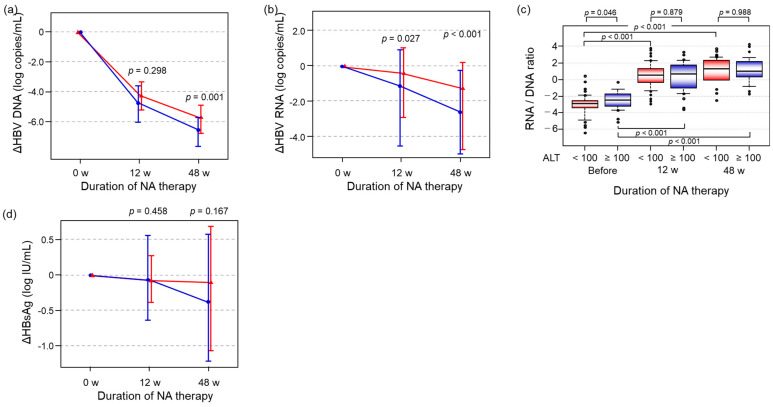
Comparison of HBV-related markers during NA therapy based on the initial ALT level. Red lines indicate the ALT low group, while blue lines indicate the ALT high group. (**a**,**b**) HBV DNA and HBV RNA decline from baseline (before NA therapy) was calculated and compared between the ALT high (N = 53) and ALT low groups (N = 33). (**c**) The RNA/DNA ratio at each time point was compared between the ALT high and ALT low groups. (**d**) HBsAg decline from baseline was calculated and compared between the ALT high and ALT low groups. All statistical analyses were performed using the Mann–Whitney U test, except (**c**), which used the Kruskal–Wallis test. Post hoc pairwise comparisons were conducted with the Steel–Dwass procedure. NA, nucleoside analog; HBeAg, hepatitis B e antigen; HBsAg, hepatitis B surface antigen.

ALT is a clinical indicator of active hepatitis and immune-mediated hepatic inflammation. It is acknowledged that such activity may modify the reductions in viral markers that occur during treatment. The study patients were divided into two groups based on baseline ALT level. Thirty-three patients whose ALT levels were <100 U/L were assigned to the ALT low group, and the remaining 53 patients whose ALT levels were ≥100 U/L were assigned to the ALT high group. Although HBV DNA and HBV RNA titers in the ALT high group declined significantly more by 48 weeks than those in the ALT low group (*p* = 0.001, *p* < 0.001, respectively) (Figure 5a,b), there was no difference in HBsAg decline between the two groups (*p* = 0.167) (Figure 5d). Although RNA/DNA ratio in the ALT low group was significantly lower than that in ALT high group (*p* = 0.048), the ratios at 12 and 48 weeks of treatment did not differ between the high and low ALT groups (*p* = 0.897, *p* = 0.988, respectively) (Figure 5c).

## 3. Discussion

HBV particles containing HBV RNA are secreted into the blood in HBV carriers regardless of whether the patient is undergoing antiviral therapy. However, the amount of RNA particles is less than 1/100 that of complete HBV particles in patients who are not undergoing antiviral therapy ([16] and Figure 3c), making it technically challenging to quantify serum HBV RNA levels accurately. Therefore, we have previously measured the sum of HBV DNA and HBV RNA (HBV DNA + RNA) by real-time PCR [17,20,25] and demonstrated the usefulness of HBV DNA + RNA measurement for predicting HBV DNA rebound after the cessation of NA therapy [20]. However, the association between serum HBV RNA level and clinical status before starting antiviral therapy has not been fully analyzed. In the present study, we measured serum HBV RNA levels using the Cobas HBV RNA real-time quantitative RT-PCR assay and examined the utility of HBV RNA for evaluating clinical status and antiviral effects by NA therapy.

First, we analyzed the correlation between serum HBV RNA levels and other HBV-related markers. HBV RNA level was significantly correlated with HBsAg, HBV DNA, and HBcrAg (Figure 1). Considering that serum samples were collected from patients before starting antiviral therapy and that HBsAg, HBV DNA, and HBcrAg are HBV replication products, these correlations suggest that HBV RNA may be useful for monitoring intracellular HBV replication activity. On the other hand, we usually observe a decline in serum HBV DNA levels as the degree of liver fibrosis progresses [26,27,28,29]. In fact, the present results also demonstrate that HBV DNA levels in patients with fibrosis stage 4 (liver cirrhosis) were significantly lower than in patients with fibrosis stages 0–3 (Figure 2a). Interestingly, as shown in Figure 2b, serum HBV RNA levels also tended to decline with liver fibrosis progression, but the decline was not significant. Therefore, progression of liver fibrosis might lead not only to inactivation of HBV replication but also to a reduction in the production of complete HBV particles containing reverse-transcribed HBV DNA genomes.

Second, we analyzed alterations in HBV-related markers during NA therapy. When we compared the alterations between HBeAg-positive and -negative patients, HBV DNA and HBV RNA levels declined during NA therapy (Figure 3a,b). However, there was no significant difference between the HBeAg-positive and -negative groups in terms of HBV DNA and HBV RNA decline after 48 weeks of NA therapy (*p* = 0.114, *p* = 0.656, respectively). Generally, the time required for HBV DNA level to decline to an unmeasurable level in HBeAg-negative patients is less than in HBeAg-positive patients [26,27,28,29]. Although our findings did not specifically support this point, we note a potential source of bias, as patients having an HBV DNA level more than 5.0 log IU/mL were selected for the study. On the contrary, the RNA/DNA ratio increased during NA therapy, and the ratio was significantly higher in HBeAg-positive patients than in HBeAg-negative patients at each of the three time points (Figure 3c). These results indicate that HBV replication activity in HBeAg-positive patients was significantly higher than in HBeAg-negative patients and suggest that HBV replication activity in HBeAg-negative patients might be suppressed to a greater extent during NA therapy in these patients. Furthermore, since HBsAg was reduced parallel to HBV RNA reduction in HBeAg-positive patients, HBV RNA might reflect not only cccDNA replication but also the amount of integrated HBV DNA.

We also compared alterations in HBV-related markers with respect to ETV versus TAF therapy. Based on several clinical studies, the effect of HBV DNA reduction might be similar between ETV and TAF therapy [30,31,32,33]. In the present study, HBV DNA reduction was similar between the two groups until 12 weeks of NA therapy. However, HBV DNA was significantly reduced in the TAF group after 48 weeks of therapy (Figure 4a), while HBV RNA was significantly reduced after 12 weeks of therapy (Figure 4b) compared to the ETV group. Although the number of patients in the TAF group was too small to draw conclusions, TAF therapy might suppress HBV replication more strongly than ETV therapy. However, the RNA/DNA ratio at each time point was similar between the ETV and TAF groups and increased after 12 weeks of therapy (Figure 4c). These results suggest that the production of HBV particles containing HBV DNA or pre-genomic RNA could be suppressed to a greater extent by TAF therapy than by ETV therapy, but the ability to suppress reverse transcription from pre-genome RNA to genomic DNA in hepatocytes might be similar between ETV and TAF therapy. Previously, Murata et al. reported that acyclic nucleoside phosphonates, including tenofovir disoproxil fumarate (TDF), could induce interferon (IFN)-λ3 in the gastrointestinal tract, but IFN-λ3 was not induced by ETV [34]. Although there is no report about an association between TAF therapy and IFN-λ3 induction, considering that TAF and TDF act in hepatocytes after being metabolized to tenofovir and that IFN-λ3 has antiviral effects on HBV infection [35], IFN-λ3 might be induced by TAF therapy, leading to additional reduction in HBV DNA and HBV RNA compared to ETV therapy.

Finally, to account for the impact of host immune responses on antiviral effects, we compared HBV DNA and HBV RNA alterations in patients with high or low baseline ALT levels. In line with our hypothesis that HBV-related markers might be more likely to decline in patients with an active immune response (high ALT group), HBV DNA and HBV RNA in the high ALT group were significantly lower after 48 weeks of therapy than those in the low ALT group (Figure 5a,b). On the other hand, the RNA/DNA ratio at each time point did not differ, regardless of ALT level (Figure 5c). Therefore, we consider that it may be difficult to regulate the transcriptional activity of cccDNA if we regulate the production of HBV viral particles.

Several HBV markers, including HBsAg, HBcrAg, and HBV RNA, can be used to evaluate the status of HBV infection and antiviral effect. Although the quantification of HBsAg and HBcrAg is commonly used clinically, and results can be obtained within 1 h, these HBV markers have limitations. HBsAg might be produced not only from cccDNA but also from HBV DNA integrated into host chromosomes [10]. Therefore, it is difficult to evaluate HBV replication activity from cccDNA by HBsAg alone. On the other hand, although HBcrAg can predict remaining intrahepatic cccDNA levels during NA therapy [36,37], HBcrAg contains HBeAg, and the value could be altered by HBeAg seroconversion [38]. Therefore, it is necessary to stratify patients based on HBeAg status. Considering these points, it is advantageous that HBV RNA is produced from cccDNA alone and is not affected by HBeAg status, providing a more direct way to evaluate the replication activity of cccDNA. Although confirmation studies using a larger sample size are needed, we propose that HBV RNA is a promising surrogate marker for monitoring HBV replication in the liver.

There were several limitations in this study. The number of study patients, especially in the TAF group, was small, and the observation period was short. Although we attempted to increase the number of enrolled patients, most of the patients in our hospital switched from ETV or another NA therapy to TAF, and their HBV DNA levels at the start of TAF therapy were less than 3 Log copies/mL. Therefore, we were unable to enroll a sufficient number of patients for proper statistical analysis with respect to NA type. Additionally, although we attempted to analyze the association between serum HBV RNA level and HBsAg, we did not observe a significant reduction in HBsAg during the observation period. Patients who successfully achieve HBsAg loss are rare because HBsAg may be produced not only from cccDNA but also from HBV DNA integrated into the host genome, even after several years of effective ETV or TAF therapy [39,40,41,42]. Considering the expected rate of HBsAg reduction, long-term observation periods are needed to clarify the impact of HBV RNA on predicting HBsAg reduction during NA therapy. Secondly, the quantification of serum HBV RNA level was performed using stored serum samples. Although we stored patient sera at −80 °C as soon as possible after blood collection, some of the samples had been stored at −80 °C for more than 10 years, raising concerns that HBV RNA might have degraded. To address this concern, we measured HBV RNA levels using sera that had been thawed as little as possible. Recently, Ohlendorf et al. reported that serum HBV RNA level should remain stable after 11 cycles of freezing and thawing [43]. Considering that the study samples were stored at −80 °C, we are confident that our results are credible. However, as there is no evidence concerning the stability of HBV RNA during long-term storage, further analysis is needed.

In conclusion, serum HBV RNA reflects intrahepatic viral replication and may not be strongly influenced by fibrosis progression. HBV RNA decline during NA therapy differed between ETV and TAF, suggesting drug-specific effects on HBV viral dynamics.

## 4. Materials and Methods

### 4.1. Patients

Eighty-six CHB patients with baseline HBV DNA ≥ 5.0 Log IU/mL and ALT < 500 U/L who started ETV or TAF therapy at our hospital between January 2000 and 2024 were enrolled. None of the patients were co-infected with other viruses, including human immunodeficiency virus and other hepatitis viruses, nor had evidence of other liver diseases, such as autoimmune hepatitis or alcoholic liver disease. To avoid the confounding effects of alcoholic liver dysfunction, heavy drinkers who ingest more than 60 g/day ethanol were excluded. All patients gave written informed consent to participate in this study. The experimental protocol conformed to the ethical guidelines of the Declaration of Helsinki and was approved by the Hiroshima University Hospital Ethical Committee (Approval ID: E2016-0704).

Serum samples were obtained at each visit to our hospital and were stored at −80 °C until use. Serum HBV RNA was measured at baseline (before NA therapy) and again after 12 and 48 weeks of NA therapy. Biochemical and hematological tests were performed in our hospital.

### 4.2. Determination of Liver Fibrosis Stage

The liver stiffness measure (LSM) was measured by FibroScan (Echosens, Paris, France). Considering the potential for measurement inaccuracies due to subcutaneous fat thickness when using the M-probe in obese patients, the XL-probe was preferentially used for patients with obesity. Patients were placed in the supine position with the right hand at maximal abduction for right lobe liver scanning. Measurements were considered valid when there were at least 10 measurements with LSM values of ≥60% and an interquartile range of <30%. The median value of these measurements was used for analysis. Analyses of LSM were conducted based on patients with available data. Liver fibrosis stage was determined using LSM stage bands, in accordance with [35]: F0–1 < 7.3 kPa; F2 7.3–9.7 kPa; F3 9.7–11.3 kPa; F4 > 11.3 kPa. Among patients who had undergone liver biopsy, fibrosis stage was determined directly from histological findings instead of LSM values.

### 4.3. Quantification of HBV RNA Level

Circulating HBV RNA levels were measured with the Cobas HBV RNA real-time quantitative RT-PCR assay for use on the Cobas 6800 Systems (Roche) Molecular Systems [44,45]. This assay has a lower limit of quantitation of 10 copies/mL and a linear range of 10 to 109 copies/mL on armored RNA [45]. HBV RNA was considered undetectable when less than 0.5 log copies/mL.

### 4.4. Measurement of Other HBV-Related Markers

HBsAg and HBeAg levels were measured by the chemiluminescence enzyme immunoassay (CLEIA) method using a commercially available enzyme immunoassay kit (Lumipulse^®^, Fujirebio Inc., Tokyo, Japan). HBV DNA level was measured using a real-time PCR assay (COBAS^®^ TaqMan HBV Test; Roche Diagnostics, Tokyo, Japan). The detectable range for HBV DNA quantitation is 1.3 to 8.2 log IU/mL. The serum HBV core-related antigen (HBcrAg) level was measured by a CLEIA HBcrAg assay kit with a fully automated analyzer system (Lumipulse System, Fujirebio Inc., Tokyo, Japan). The detection limit for the assay was 3.0 log U/mL.

### 4.5. Statistical Analysis

Categorical variables are reported as counts, while continuous variables are expressed using the median and range. Spearman’s rank correlation test was performed to investigate the relationship between HBV RNA and the other HBV markers, such as HBsAg, HBV DNA, and HBcrAg. Univariate pairwise comparisons were performed using the Mann–Whitney U test or Fisher’s exact test. For all statistical analyses, a *p* value < 0.05 was considered statistically significant. All statistical analyses were performed using EZR version 1.68 (Saitama Medical Center, Jichi Medical University, Saitama, Japan) [46].

## Figures and Tables

**Table 1:** Clinical characteristics of study subjects.

Factors	Overall (N = 86)	ETV Group (N = 80)	TAF Group (N = 6)	*p* Value
Age (year)	48 (21–81)	49 (21–81)	40 (34–51)	0.125 *
Gender (Male/Female)	63/23	59/21	4/2	0.656 **
Platelet count (×10^4^/μL)	15.9 (5.4–37.8)	15.7 (5.4–37.8)	19.1 (13.8–21.7)	0.282 *
AST (U/L)	57 (16–935)	57 (16–304)	87 (37–935)	0.282 *
ALT (U/L)	61 (13–389)	60 (13–389)	92 (31–314)	0.282 *
HBsAg (Log IU/mL)	3.56 (−0.78–5.46)	3.54 (−0.78–5.46)	3.61 (2.78–4.99)	0.919 *
HBeAg (+/−)	47/39	43/37	4/2	0.685 **
HBV DNA (Log copies/mL)	7.2 (5.0–10.0)	7.2 (5.8–10.0)	7.8 (5.0–8.6)	0.919 *
HBV RNA (Log copies/mL)	4.35 (undet.–7.96)	4.25 (undet.–7.96)	4.73 (1.22–6.05)	0.872 *
HBV genotype (B/C/ND)	2/69/15	2/64/14	0/5/1	1.000 **
Fib-4 index	2.05 (0.47–12.29)	2.05 (0.47–9.97)	1.85 (0.83–12.29)	0.780 *
Fibrosis stage (F0-1/2/3/4/ND)	11/24/17/4/30	8/22/17/3/30	3/2/0/1/0	0.007 *

Univariate analyses were performed by Mann–Whitney U test (*) and Fisher’s exact test (**). ETV group: Patients with entecavir therapy, TAF group: Patients with tenofovir alafenamide therapy, AST: Aspartate aminotransferase, ALT: Alanine aminotransferase, HBsAg: Hepatitis B surface antigen, HBeAg: Hepatitis B e antigen.

## Data Availability

The data that support the findings of this study are available from the corresponding author upon reasonable request. The data are not publicly available due to privacy or ethical restrictions.

## Abbreviations

The following abbreviations are used in this manuscript:

HBV	hepatitis B virus
HBsAg	hepatitis B surface antigen
HBeAg	hepatitis B e antigen
HBcrAg	hepatitis B core-related antigen
cccDNA	covalently closed circular DNA
CHB	chronic hepatitis B
HCC	hepatocellular carcinoma
NA	nucleotide/nucleoside analog
ETV	entecavir
TAF	tenofovir alafenamide
AST	aspartate aminotransferase
ALT	alanine aminotransferase
